# Blue Rubber Bleb Nevus Syndrome with Long-Term Follow-Up: A Case Report and Review of the Literature 

**DOI:** 10.1155/2018/8087659

**Published:** 2018-11-25

**Authors:** Hideaki Nakajima, Hiroshi Nouso, Naoto Urushihara, Koji Fukumoto, Masaya Yamoto, Hiromu Miyake, Akinori Sekioka, Akiyoshi Nomura, Yutaka Yamada

**Affiliations:** Department of Pediatric Surgery, Shizuoka Children's Hospital, 860 Urushiyama, Aoi-ku, Shizuoka 420-8660, Shizuoka, Japan

## Abstract

Blue rubber bleb nevus syndrome (BRBNS) is a rare disease in which venous malformations (VMs) involve any body organ, most commonly the skin and the gastrointestinal (GI) tract. Treatment of BRBNS aims at preserving the GI tract as much as possible. Although there are several dozen case reports about BRBNS that describe short-term clinical courses, a few provide an account of long-term clinical course. Here, we report a case of BRBNS in a girl that required multiple abdominal surgeries due to the GI VMs and a recurrence at an interval of 14 years. The preferred approach for gastrointestinal VMs involves the complete resection of all lesions without residual VMs. It is important to bear in mind the possibility of delayed recurrence of GI VMs after surgical or endoscopic treatment.

## 1. Introduction

Blue rubber bleb nevus syndrome (BRBNS) is a rare condition, characterized by multiple venous malformations (VMs) of the skin and gastrointestinal (GI) tract [1, 2]. The GI lesions could cause abundant bleeding and lead to severe anemia. If GI bleeding becomes uncontrollable by conservative treatment, endoscopic or surgical intervention is required. In previous reports, short-term postoperative courses were mostly uneventful; however, some cases had prolonged courses owing to residual lesions or recurrence.

We report the case of BRBNS with GI lesions involving long-term follow-up and multiple surgical treatments. We also provide a brief review of the literature regarding long-term follow-up of GI lesions of BRBNS treated endoscopically or surgically.

## 2. Case Presentation

**Figure 1:**
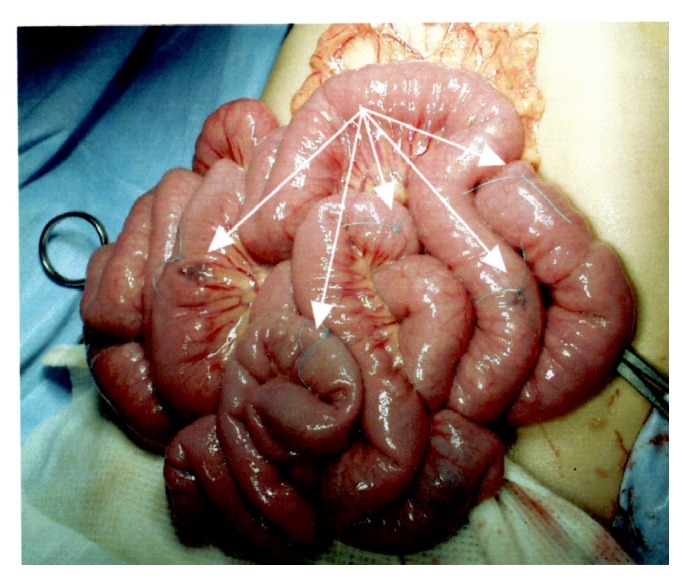
Demonstration of the first operation at 5 years of age: multiple venous malformations on the small intestine (marked by the arrows) were seen.

A 19-year-old girl was admitted to our hospital for surgery with a 15-year history of GI bleeding associated with BRBNS. She had repeated episodes of resections of hemangiolymphangiomas on the right thoracic wall from 3 months to 3 years of age. At 4 years of age, she presented with a few cutaneous small VMs on the feet; hematological examination revealed persistent anemia and positive fecal occult blood. Colonoscopy revealed a VM on the right colon. She underwent an exploratory laparotomy at 5 years of age. Multiple VMs were identified (Figure 1): 1 on the lesser curvature of the stomach, 7 on various parts of the small intestine, 1 on the hepatic flexure of the colon, and 1 on the liver. Intestinal lesions were resected, and gastric and hepatic lesions were left untreated to avoid injury to surrounding organs. The pathological finding was cavernous hemangioma, so she was diagnosed with BRBNS. Four months later, she suffered superior mesenteric artery thrombosis and underwent partial resection of the small intestine. Since then, although mild anemia persisted, it was treatable by oral iron replacement therapy, and the clinical course was uneventful for 10 years.

**Figure 2:**
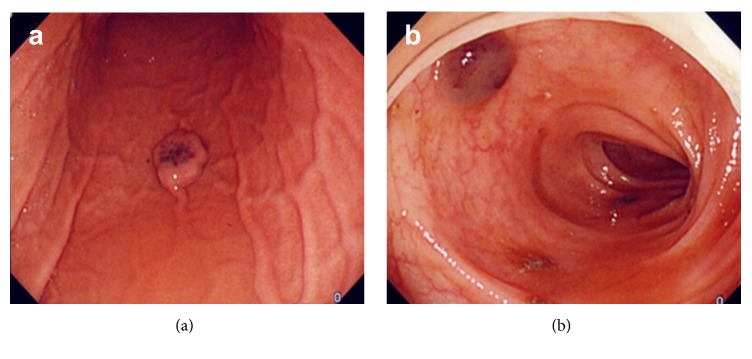
Endoscopic findings of venous malformations at 19 years of age. (a) On the posterior corpus of the stomach; (b) On the sigmoid colon.

**Figure 3:**
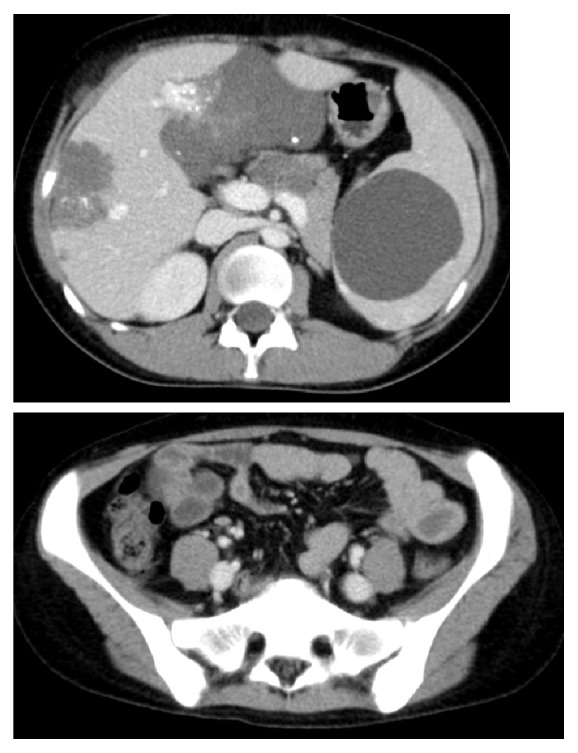
Computed tomography at 19 years of age revealed multiple VMs on almost the entire GI tract, liver, and spleen.

**Figure 4:**
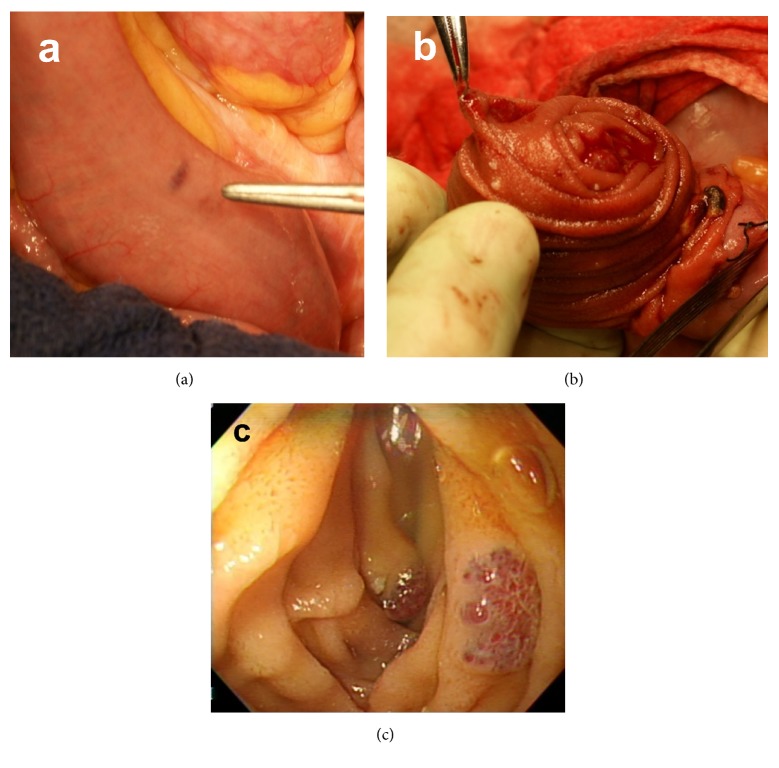
Demonstration of the second operation 19 years of age. (a) VM on the small intestine; (b) Lesions were exposed by intussuscepting the bowel through enterostomies; (c) Enteroscopy through the enterotomies was performed to detect the remaining lesions.

At 15 years of age, the anemia got slightly worse. Hematological examination revealed that Hemoglobin (Hb) level was 7–8 mg/dL. GI endoscopy, including transanal double-balloon endoscopy and capsule endoscopy, and contrast-enhanced computed tomography (CT) revealed multiple VMs on almost the entire GI tract, including the stomach, duodenum, jejunum, ileum, and colon (Figures 2 and 3). VMs were also found in the right pleural space, hepatic hilum, liver, and spleen. An additional finding was a right ovarian cyst. Anemia and GI bleeding became more severe despite the administration of a *β*-blocker since she was 18. Hematological examination revealed that Hb level was 4 mg/dL. Frequent blood transfusion was prescribed. At 19 years of age, CT revealed an increase in the size of known lesions. In order to control GI bleeding, she underwent the second surgery for GI VMs. We performed resection of all lesions on the stomach and the intestine, partial resection of the small intestine, appendectomy, splenectomy, and right ovarian cystectomy. Enteric lesions were removed through a gastrotomy, 11 enterotomies, and a sigmoid colotomy. Palpation of a lesion on the posterior gastric corpus revealed the absence of transmural involvement, so the lesion was resected above the proper muscular layer. Palpable lesions on the small intestine (Figure 4(a)) were marked with serosal sutures and were exposed by intussuscepting the bowel through enterotomies (Figure 4(b)). For transmural lesions, wedge resection was performed. For the lesions grossly localized in the mucosal layer, small or polypoid lesions were ligated and resected, and elevated lesions were treated with argon plasma coagulation (APC). Intraoperative endoscopy was performed through enterotomies to detect the remaining lesions (Figure 4(c)). The number of the lesions on the small intestine was > 350. Localized dilatation of the small intestine caused by adhesion of the mesentery was found and resected partially to avoid involvement of an adhesive bowel obstruction. A palpable lesion on the sigmoid colon was ligated and resected through colotomy. Although preoperative colonoscopy revealed several other small lesions from the cecum to the sigmoid colon, they were left untreated because colonoscopic treatment would be possible at a later date. The splenic lesion was suspected to invade the tail of the pancreas, so the pancreatic parenchyma was also resected with splenectomy. Operative duration was 11.5 hours. Intraoperative blood loss was 325 mL. Postoperative pancreatic fistula was observed but successfully managed by conservative treatment. Pathology of surgical specimens revealed VMs in the stomach, small intestine, sigmoid colon, and spleen. The splenic lesion was found to invade pancreatic tissue. The right ovarian cyst was found to be a mature teratoma. Postoperative GI endoscopies revealed the remaining VMs on the duodenum and colon. The patient had no bleeding or anemia for 5 years after the surgery.

## 3. Discussion

BRBNS is a rare disease characterized by multiple VMs in the skin and the GI tract [1, 2]. The characteristic cutaneous lesions consist of deep blue, rubbery blebs, which are easily compressible. Other organs affected include the central nervous system, joints, liver, spleen, lungs, eyes, and urinary tract. If a person has a cutaneous angioma associated with another symptom such as melena, epilepsy, hemoptysis, hematuria, paralysis, or visual disorder, the possibility of BRBNS should be considered. Gastrointestinal and radiological examinations such as endoscopy, CT, and MRI are the main tools for diagnosis. The differential diagnosis of GI hemangiomatosis includes BRBNS, Osler–Weber–Rendu syndrome, Maffucci syndrome, and Klippel–Trenaunay–Weber syndrome [2].

Treatment of BRBNS is directed to the symptom. Gastrointestinal lesions may manifest clinically with chronic continual bleeding. Conservative treatment by oral iron supplementation or blood transfusion is usually sufficient. Pharmacologic treatment to control GI bleeding with agents such as corticosteroids, interferon-*α*, and *β*-blocker has been attempted [1, 13, 14], but there is no convincing evidence of durable beneficial effects of any drug treatment. Recently, the mammalian target of rapamycin (mTOR) inhibitor sirolimus has been reported to be effective in reducing VMs related to BRBNS [13, 14]. If surgical intervention is considered, it is important to preserve as much of the GI tract as possible. In some previous reports, resections of long segments were required because of intussusceptions or extensive areas of involvement [15, 16]. Surgical wedge resection and hemangioectomy are reasonable options to control GI bleeding [17]. Combined intraoperative endoscopy is recommended to avoid oversight of small lesions and to avoid unnecessary bowel resection. Endoscopic treatments such as polypectomy, band ligation, clipping, and APC have been reported recently but are controversial. Some investigators describe endoscopic polypectomy or band ligation as involving high risk of perforation or ulceration in patients with transmural lesions. They are convinced that such lesions should be managed by full-thickness wedge resection [11]. We performed ligation or APC for GI VMs localized in the mucosal layer and wedge resection for transmural lesions with no such complications. Endoscopic electrocautery or APC seemed to be useful in treating small VMs; however, surgical resection seemed to be safe for the treatment of transmural large VMs.

There are 91 cases of BRBNS with GI VMs reported in the literature identified from MEDLINE [18]. However, most of those that required endoscopic or surgical treatment for GI VMs and/or related bleeding concerned only first interventions, with either no follow-up or short-term follow-up for 6 months at most. We analyzed cases with short- to long-term follow-up for > 1 year after the initial endoscopic or surgical treatment. There was no recurrence in the cases with short follow-up periods (1–3 years). Conversely, cases with longer follow-up periods (>5 years) included unstable postoperative courses (Table 1) [3–12]. Such cases with needs of iron supplementation or blood transfusion might have VM lesions left over on the GI tract, causing bleeding (for example, cases 1, 3, and 7). These cases should be closely followed up. There were cases with stable postoperative periods for 5 years after the first treatment (cases 2, 6, 8, 9, and 10); however, some of these cases revealed recurrence and required a second treatment (cases 2 and 6). Our present case also followed a relatively stable clinical course after the first operation, but revealed mild anemia resulting in a second series of bleeding episodes at an interval of 10 years. This case suggests that long-term follow-up is necessary in BRBNS patients even if the early postoperative course is uneventful. Regular imaging studies like CT and endoscopy would be recommended in the course of follow-up. If VMs on the GI tract recur and the bleeding from the lesions occurs again hereafter, the treatment aims at preserving the GT tract as much as possible. Medical management would have priority over the surgical bowel resection.

In conclusion, we report a case of BRBNS that required a second surgery for delayed recurrence. Long-term follow-up is important in the BRBNS patients following even successful resections.

## Figures and Tables

**Table 1:** Review of cases of blue rubber bleb nevus syndrome with venous malformations on the gastrointestinal tract.

No.	Year	Sex	Age at onset	Age at treatment	Sites of lesions	Treatment	Outcome	follow up period from the 1st / last treatment (year)
1	2001 [3]	F	0	0	—	right colectomy	—	37 / —
				0–6	—	partial gastrectomy, small bowel resection, total colectomy	blood transfusions required	
				34–37	small intestine, rectum	endoscopic sclerotherapy	oral iron, vitamin and caloric supplementation	

2	1996 [4]	F	6	7	intestine	surgical resection	stable for 5 years	25 / 5
				13	small intestine, colon	surgical resection	stable for 5 years	
				26	duodenum, ileum, colon	surgical resection	stable for 5 years	

3	1997 [5]	M	5	9	stomach	endoscopic electrocautery	oral iron supplements and blood transfusions required	21 / 1
				23	stomach, duodenum, small intestine, colon	intraoperative endoscopic polypectomy, surgical resection	stable for 1 year	

4	1990 [6]	F	9	9	whole GI tract	endoscopic sclerotherapy (for the upper and lower GI)	GI bleeding continued	13 / short period
				15	whole GI tract	surgical resection	stable for 4 years	
				19	upper and lower GI tract	intraoperative endoscopic electrocautery, surgical resection	stable for the short period	

5	2013 [7]	F	1	12	stomach	surgical resection	—	13 / 3.5
				22	colon	endoscopic ligation and sclerotherapy	stable for 3.5 year	

6	2005 [8]	F	19	19	colon	endoscopic clipping	stable for 5 years	7 / short period
				25	small intestine, colon	endoscopic clipping and polypectomy, surgical resection	stable for the short period	

7	2009 [9]	M	0	2	stomach, colon	endoscopic electrocautery (for the stomach)	blood transfusions required for 4 years	7

8	2003 [10]	F	0	0	colon	endoscopic APC	stable for 5 years	5

9	2007 [11]	F	8	12	stomach, small intestine, colon	Surgical wedge resection, intraoperative endoscopic sclero-coagulation	stable for 5 years	5

10	2010 [12]	M	4	4	stomach, colon	endoscopic sclerotherapy	—	5 / 3
				6	small intestine	surgical resections through enterotomies	stable for 3 years	

11	Our case	F	4	5	stomach, small intestine, colon	surgical resection	stable for 10 years with oral iron supplementation	19 / 5
				19	stomach, duodenum, small intestine, colon	surgical resection, APC	stable for 5 year	

“—” means no data described; GI: gastrointestinal; APC: argon plasma coagulation.
